# Extracorporeal Membrane Oxygenation in Massive Pulmonary Hemorrhage Secondary to Arteriovenous Malformation

**DOI:** 10.7759/cureus.100315

**Published:** 2025-12-29

**Authors:** Maria Vidal, Joana Nogueira, Andreia Santos, Ricardo Freitas, Eduardo Sousa

**Affiliations:** 1 Intensive Care Medicine, Unidade Local de Saúde de Coimbra, Coimbra, PRT

**Keywords:** arteriovenous malformation, ecmo, extracorporeal membrane oxygenation, massive hemoptysis, pulmonary av fistula

## Abstract

We report a case of a 63-year-old man with severe pulmonary hemorrhage secondary to arteriovenous malformation and ventilatory failure due to reduced alveolar diffusion and airway obstruction. Bronchoscopy revealed high-flow active bleeding and clots obstructing the orotracheal tube and bronchial tree, refractory to local therapeutic measures. The patient was successfully supported with venovenous extracorporeal membrane oxygenation (ECMO) without systemic anticoagulation for 12 days as a bridge to definitive therapy. During this period, two angiographic arterial embolization attempts were unsuccessful, ultimately necessitating a right lower pulmonary lobectomy. In cases of massive hemoptysis, maintenance of airway patency and bleeding control should be carried out simultaneously. Treatment often includes bronchoscopy, angiographic arterial embolization, and, in the case of refractory bleeding, surgery. Venovenous ECMO provides short-term stabilization in pulmonary hemorrhage with refractory hypoxemic respiratory failure that is limiting a definitive intervention to achieve hemostasis.

## Introduction

Pulmonary arteriovenous fistulas are rare vascular malformations. They represent direct communications between arteries and veins without any intervening capillary bed, creating a right-to-left anatomical shunt. They are most associated with hereditary hemorrhagic telangiectasia, but there are also sporadic arteriovenous malformations (AVMs), or more rarely, acquired after infections or trauma. While pulmonary AVMs most commonly present with dyspnea, hypoxemia, or paradoxical embolic events, hemoptysis is an infrequent manifestation, and massive hemoptysis is particularly uncommon. When present, bleeding is thought to result from rupture of fragile, high-flow vascular channels, posing a life-threatening risk. Owing to their low incidence and atypical presentation, pulmonary AVMs are an often-overlooked cause of massive hemoptysis, yet recognition is crucial, as timely diagnosis and appropriate intervention - either endovascular or surgical - can be life-saving [1-5].

The standard treatment for most AVMs is angiographic intervention. The main advantages of this technique are that it is less invasive than surgical intervention and can easily be performed multiple times. Many AVMs have more than one afferent artery, each requiring embolization to completely exclude the lesion. While surgical excision has the benefit of offering definitive treatment for a single lesion, it is more invasive than angiographic embolization. If angiographic embolization fails and is contraindicated or unavailable, conservative surgical excision is the procedure of choice, as thoracotomy allows control of hilar vessels, and the fistula can be excised without the need for pulmonary parenchyma resection [1,6,7].

Extracorporeal membrane oxygenation (ECMO) is used in cases of acute cardiac and/or respiratory failure of reversible cause (or, when irreversible, as a bridge to transplant) when conventional treatment and support fail [8-15]. The use of venovenous (VV) ECMO in adults is rapidly increasing worldwide, with more than 24.000 cases reported due to respiratory failure in adults recorded by 2020, according to data from the Extracorporeal Life Support Organization (ELSO) [8]. Although hemorrhage is often considered a relative contraindication to ECMO, it may be lifesaving in selected cases of pulmonary hemorrhage and represents one of the specific clinical scenarios in which its use can be justified [8]. However, only a limited number of cases have been reported in the literature, and experience remains scarce, particularly regarding the use of ECMO without systemic anticoagulation.

## Case presentation

A 63-year-old man presented to the Emergency Department of his local hospital with a productive cough and hemoptysis. The patient had a history of hypertension, for which he was on medication, and a previous history of smoking (estimated 10 pack-years).

**Figure 1:**
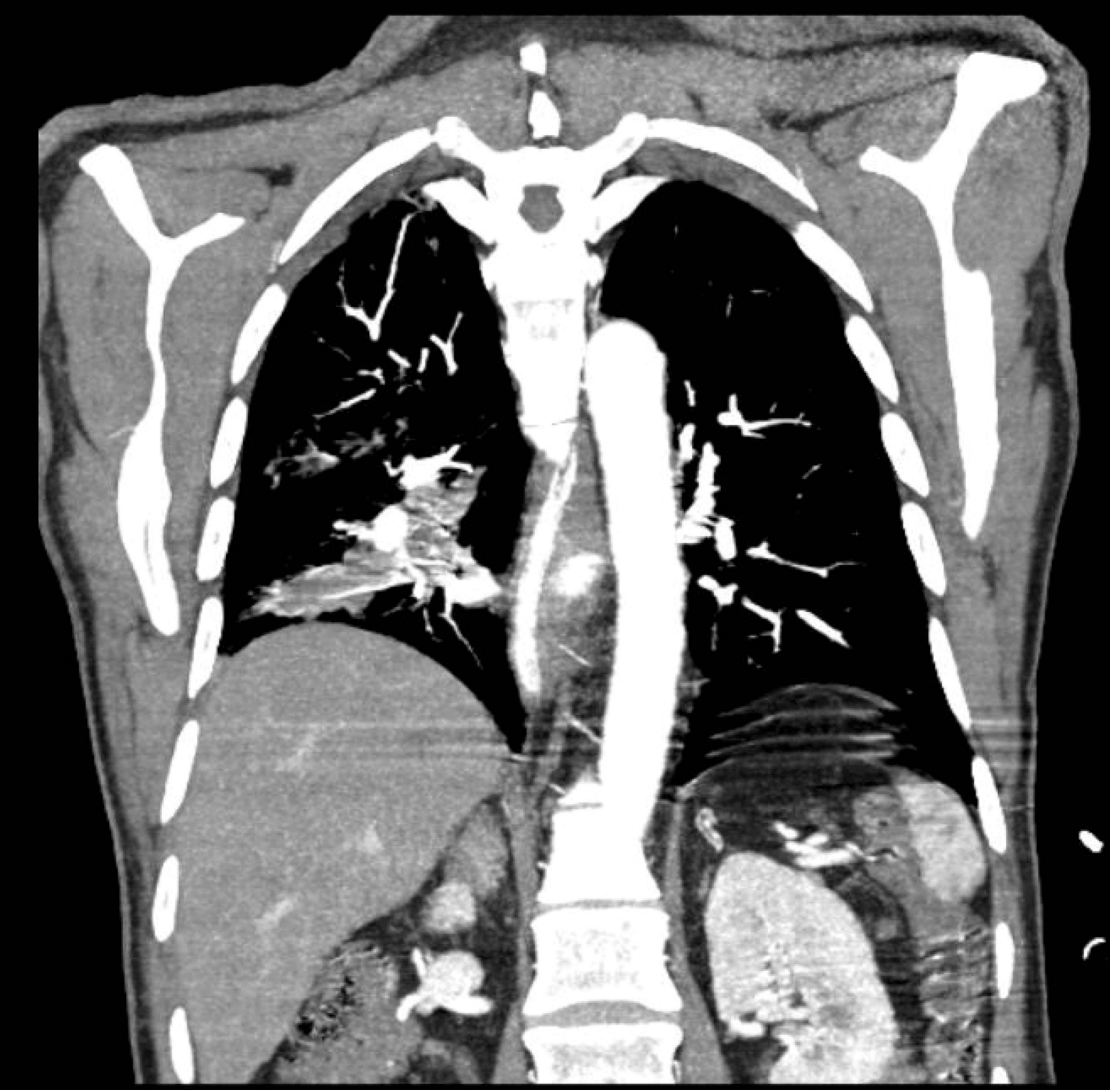
CT showing a lower right lobe 32 mm lesion

There was a history of a recent hospitalization in the Pulmonology Department to clarify the cause of previous episodes of hemoptysis, during which a 32 mm lesion was observed in the right lower lobe on thoracic tomography (Figure 1). Bronchoscopy was performed, without evidence of endobronchial lesions, and biopsies revealed tissue with a chronic inflammatory appearance of nonspecific nature. During this hospitalization, the patient did not have other episodes of hemoptysis and was discharged home.

He was conscious and cooperative, had no respiratory insufficiency, no drop in hemoglobin level, and no other analytical abnormalities. During transfer to radiology, the patient presented with massive hemoptysis, requiring orotracheal intubation, mechanical ventilation, and subsequent admission to the Intensive Care Unit. Bronchoscopy revealed diffuse hemorrhage, limiting the visualization of the bronchial tree, even after aspiration of multiple clots.

On the sixth day of hospitalization, the patient had recurrent hemorrhage, compromising ventilation due to both reduced alveolar diffusion capacity and obstruction of the distal part of the orotracheal tube by clots. Given the severe respiratory insufficiency of reversible cause, the Intensive Care Unit of our center was contacted, and the ECMO team was activated.

Our mobile ECMO team initiated VV ECMO in the referring hospital after cannulation of the right internal jugular and femoral veins. Due to persistent hemorrhage, anticoagulation of the circuit was not initiated. Subsequently, the patient was airlifted to our center, accompanied by the ECMO team, and the transport proceeded without incident.

Upon arrival, with the support of the Pulmonology Department, the patient underwent rigid bronchoscopy, visualizing a clot completely occluding the right bronchial tree and partially the left. After partial clot removal in the basal segments of the right lower lobe bronchus, continuous high-output hemorrhage was observed, flooding the entire tracheobronchial tree despite continuous aspiration, with no response to topical measures (topical adrenaline, cold saline, and procoagulant paste application). Aminocaproic acid was administered and terlipressin initiated, but no clinical response was observed. Reintubation via orotracheal tube was performed, with an attempt at selective intubation of the left main bronchus, which was unsuccessful.

**Figure 2:**
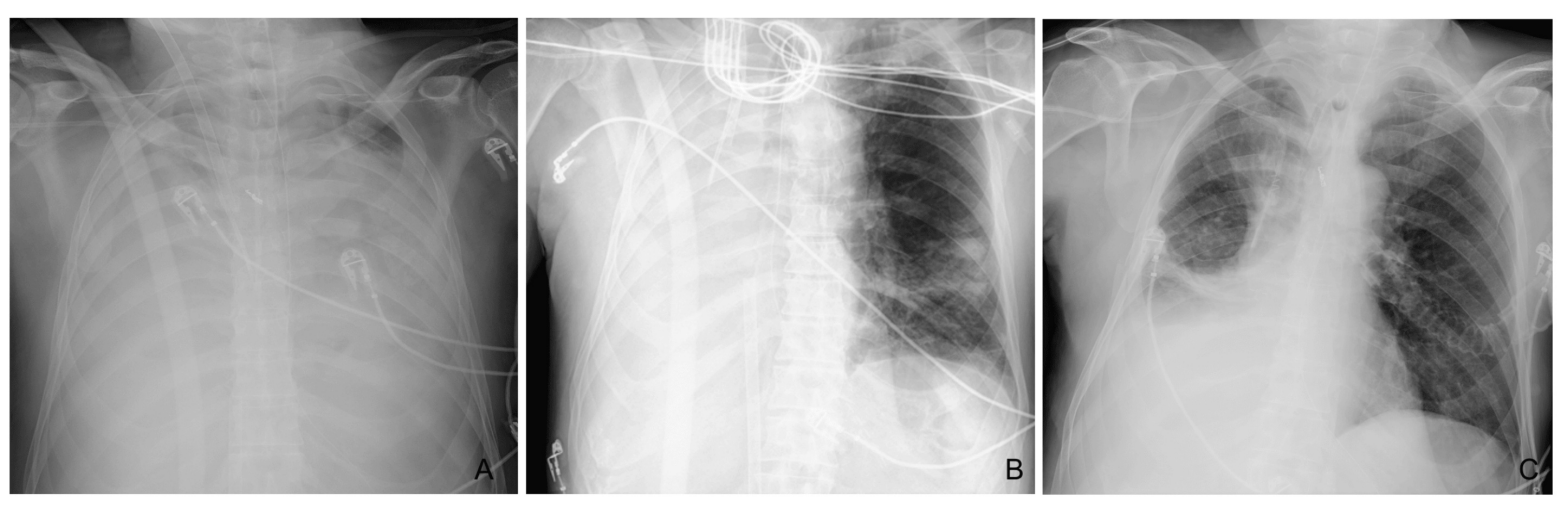
Chest radiograph showing (A) total opacification of both lung fields, (B) two days after right lower lobectomy, and (C) at the time of transfer

Angiography revealed active hemorrhage from the right bronchial artery, and embolization was performed with microcoils and polyvinyl alcohol (PVA) particles. About 24 hours later, the patient had recurrent hemorrhage, and angiography once again showed signs of active hemorrhage from a lower branch of the right bronchial artery, despite previous embolization. A second embolization was performed, with successful exclusion of the bleeding vessel at the end of the procedure. At this time, chest X-ray showed total opacification of both lung fields (Figure 2A).

**Figure 3:**
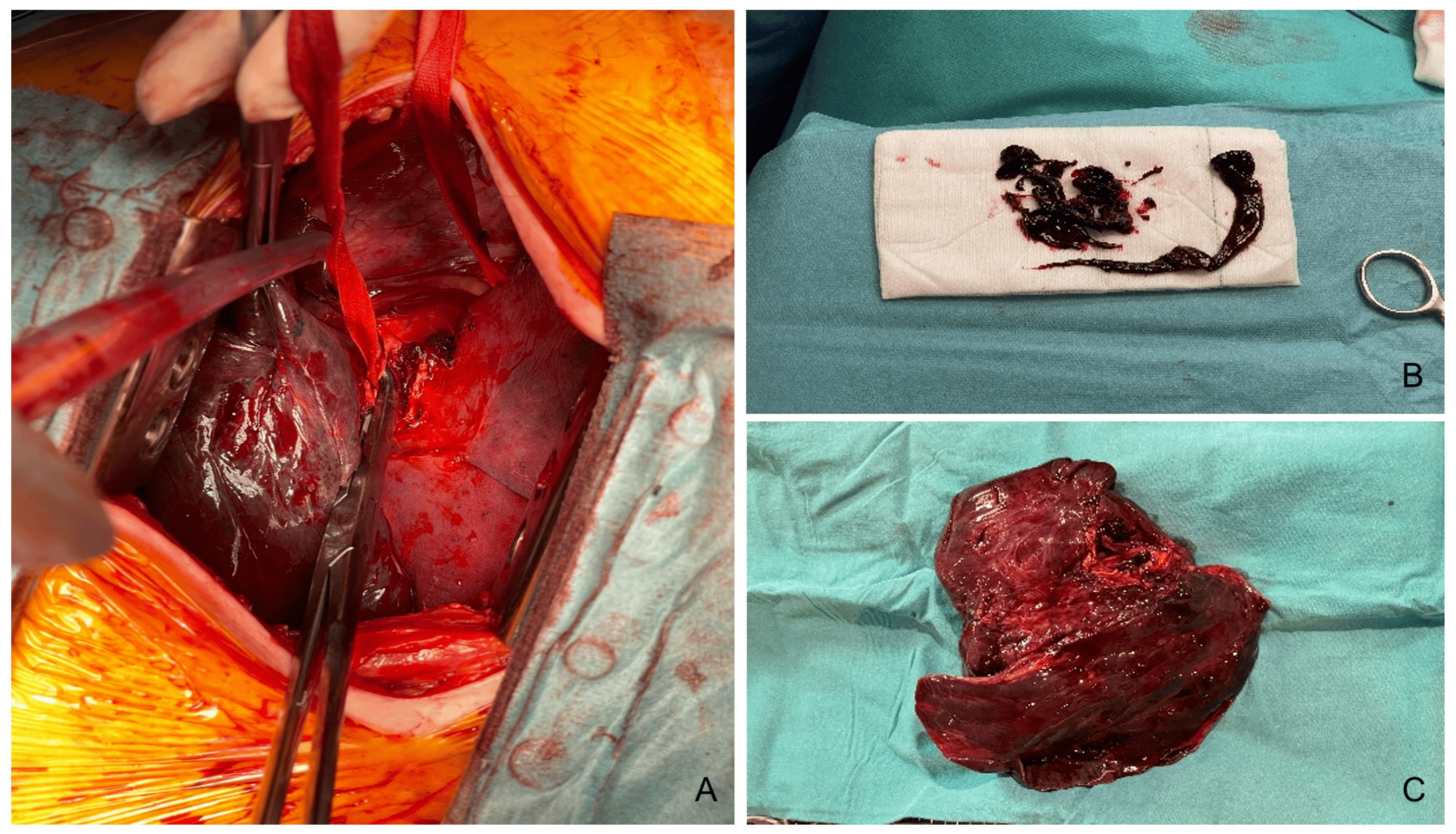
Posterolateral thoracotomy showing (A) the right lower lobe and right inferior bronchus, (B) blood clots being removed from the right bronchus, and (C) the surgical specimen of the lower right lobe

Due to persistent, although reduced, hemorrhage, thoracic surgery was contacted, and urgent surgical intervention was decided. Following right postero-lateral thoracotomy and entry into the pleural cavity, an area of lighter color than the adjacent lung, filled with aged clots, was identified, with no palpable nodular area. The afferent arterial branches to the right lower lobe at the fissure level were identified and ligated. The right lower lobe bronchus was divided, and bronchial tree aspiration up to the origin of the right upper lobe bronchus was performed (Figure 3C).

During the surgical procedure, bronchoscopy was performed for bronchial tree aspiration (Figure 3B). The patient remained on ECMO, under neuromuscular blockade, with tidal volumes between 10 and 30 mL. Lung recruitment was attempted, reaching insufflation pressures around 35 mmHg, but was unsuccessful, and the lung remained collapsed. Histology of the surgical specimen identified an arteriovenous malformation, with multiple thrombi and intrabronchial blood clots, besides areas of epithelial pulmonary remodeling.

One day after surgical intervention, the patient showed progressive gain in tidal volume, around 200-400 mL, and good response to alveolar recruitment maneuvers. Chest X-ray showed aeration of the left lung (Figure 2B).

The patient developed edema of the right upper limb, and Doppler revealed thrombosis of the right jugular vein just below the cannula. For this reason, therapeutic anticoagulation with low molecular weight heparin was started.

Multiple bronchoscopies were performed on various occasions, with clot aspiration and successive re-permeabilization of the main left bronchus and right lobar bronchi. On the seventh postoperative day, chest X-ray showed aeration of the upper part of the right lung field, besides improvement on the left. Given the progressive improvement, ECMO weaning and discontinuation were possible on the 12th day. The patient was extubated on the 27th day of invasive mechanical ventilation. However, due to secretion management difficulty and respiratory difficulty with accessory muscle use, reintubation was necessary, and surgical tracheostomy was performed.

After stabilization, mechanical ventilation was progressively weaned, and the patient was transferred back to the hospital of origin, 32 days after the initial hospitalization. On the 44th day, tracheostomy decannulation was possible, and one week later, the patient was discharged to a convalescence unit for continuation of motor rehabilitation, with peripheral saturation ranging between 96% and 98% in room air, without significant desaturation during exercise.

## Discussion

The authors describe a case of a patient with an inaugural massive hemoptysis where simultaneous management of airway patency and hemorrhage control was crucial. Subsequently, priority should be given to definitive treatment, such as arterial embolization or surgery.

Historically, therapeutic options for managing these patients have been limited, resulting in high mortality rates when conservative treatment is chosen. For this reason, surgery assumed a predominant role in its treatment. However, when the volume of hemoptysis is such that emergency hemostasis is unachievable, and airway patency is compromised due to flooding, the patient may not be a candidate for angiographic embolization or surgery. Hence, the necessity of development of new techniques to sustain life until definitive treatment is achieved [16-19].

Airway stabilization and hemorrhage isolation

Airway stabilization and hemorrhage isolation should be prioritized. If the bleeding side is known, the patient should be positioned laterally with the bleeding side down to prevent hemorrhage from flooding other lung areas. Orotracheal intubation is recommended, if possible, using a tube with a diameter that allows the passage of flexible bronchoscopes with working channels for clot extraction and for introducing bronchial blockers. Selective intubation of one of the main bronchi can be attempted to isolate the hemorrhagic lung from the contralateral lung. Double-lumen endotracheal tubes are designed to isolate each lung and are commonly used in thoracic surgery; however, their use is almost obsolete in cases of massive hemoptysis since their individual lumens are narrow and do not allow for flexible bronchoscopes capable of aspirating clots [20-24].

Flexible and rigid bronchoscopy

Flexible bronchoscopy plays a critical role in the management of patients with massive hemoptysis. Due to its versatility, it can be advantageous in various situations, such as determining the site of bleeding, assisting with selective intubation, introducing bronchial blockers, extracting clots, or for therapeutic purposes [20,21,25,26], allowing direct instillation of drugs into bleeding segments.

On the other hand, rigid bronchoscopy offers some advantages over flexible bronchoscopy in the management of patients with massive hemoptysis [27]. The rigid bronchoscope provides better airway stabilization by allowing immediate selective isolation of each main bronchus and simultaneous ventilation [28]. It also facilitates the extraction of large clots obstructing the airway.

Flexible and rigid bronchoscopies were of utmost importance in managing the patient described by the authors. Rigid bronchoscopy was used in an attempt to stop the bleeding, while flexible bronchoscopy was performed almost daily during the first few days to allow optimal airway clearance.

Venovenous extracorporeal membrane oxygenation (VV ECMO)

VV ECMO was used as an advanced rescue option for this selected patient, with hemoptysis causing severe airway obstruction. In this context of refractory hypoxemic respiratory failure that limits definitive intervention to achieve hemostasis, VV ECMO can enable short-term patient stabilization [29,30-32].

Active hemorrhage or increased bleeding risk often constitute relative contraindication to ECMO, as its use requires systemic anticoagulation to maintain circuit patency and prevent systemic thromboembolism. Risk-benefit analysis is essential in cases of massive hemoptysis. Some authors hypothesize that newer ECMO technology does not require the same levels of anticoagulation as in the past, allowing for acceptably lower activated thromboplastin times [33]. In this case, the ECMO team’s extensive experience was essential for safely managing the circuit without anticoagulation, which demands meticulous monitoring of the circuit for clot formation, optimal flow maintenance, and careful management of oxygenator function and line integrity.

There are other reported cases of successful VV ECMO use in patients with hemoptysis and refractory respiratory failure. The causes of hemorrhage varied and primarily include cases of diffuse alveolar hemorrhage secondary to other pathologies [15,32,34-38]. Cao et al. reported in 2014 the use of ECMO in a case of massive hemoptysis due to rupture of a bronchial artery aneurysm, where the patient underwent embolization and later required lobectomy due to hemorrhage recurrence [39]. Hsu used ECMO in a case of hemoptysis originating from a fistula between the phrenic artery and the left bronchial artery, successfully treated after angiographic embolization [30].

Arterial embolization and surgery

After airway stabilization and initial hemostatic measures, most patients require definitive treatment, often involving percutaneous arterial embolization.

Arterial embolization is minimally invasive and highly effective in controlling hemoptysis [40]. In the case described, the patient underwent multiple arterial embolizations, which, although successful in excluding the culprit vessel, were not entirely effective in stopping the bleeding and ultimately led to the decision to proceed with surgery. The indications for surgery, previously considered the only available treatment for massive hemoptysis, have evolved with the development of other techniques. However, complex arteriovenous malformations, iatrogenic rupture of the pulmonary artery, or refractory hemoptysis remain circumstances where surgery should be considered as first-line treatment [21,26,41,42].

## Conclusions

In cases of AVMs, the main goal is the interruption of flow to the malformation. Although ECMO use in cases of pulmonary hemorrhage is controversial, in our case, given the recurrent hemorrhage and associated hypoxemia, refractory to conventional measures (endoscopic treatment, systemic treatment), ECMO allowed patient stabilization while simultaneous control of the hemorrhage and intervention with curative intention was performed. The main factors influencing the decision to initiate ECMO included the severity of hemorrhage, the lack of response to conservative measures, the patient’s clinical condition, and the experience of the team in managing the ECMO circuit.

This case highlights the importance of a multidisciplinary approach in the treatment of patients with massive hemoptysis and severe respiratory failure, including the use of ECMO. This is especially true in the age of modern ECMO technology, where the circuit can be maintained without systemic anticoagulation.
